# IDH mutations in G2-3 conventional central bone chondrosarcoma: a mono institutional experience

**DOI:** 10.1186/s12885-023-11396-y

**Published:** 2023-09-26

**Authors:** Elisabetta Setola, S. Benini, A. Righi, G. Gamberi, E. Carretta, C. Ferrari, S. Avnet, E. Palmerini, G. Magagnoli, M. Gambarotti, P. L. Lollini, M. Cesari, S. Cocchi, A. Paioli, A. Longhi, K. Scotlandi, M. A. Laginestra, D. M. Donati, N. Baldini, T. Ibrahim

**Affiliations:** 1 Osteoncology, Bone and Soft Tissue Sarcomas and Innovative Therapies, IRCCS Istituto Ortopedico Rizzoli, Via Pupilli 1, Bologna, 40136 Italy; 2 Department of Experimental, Diagnostic and Specialty Medicine (DIMES), University of Bologna, Bologna, Italy; 3 Department of Pathology, IRCCS Istituto Ortopedico Rizzoli, Bologna, Italy; 4 Department of Biomedical and Neuromotor Sciences, University of Bologna, Bologna, Italy; 5 IRCCS Istituto Ortopedico Rizzoli, Bologna, Italy; 6 Experimental Oncology Laboratory, IRCCS Istituto Ortopedico Rizzoli, Bologna, Italy; 7 Biomedical Science and Technologies Unit, IRCCS Istituto Ortopedico Rizzoli, Bologna, Italy

**Keywords:** Chondrosarcoma, *IDH* mutation, Prognostic factors, cancer metabolism, Sarcoma, Bone sarcoma, Target therapy, IDH inhibitors

## Abstract

**Background:**

Heterozygous isocitrate dehydrogenase (*IDH*) mutations occur in about half of conventional central bone chondrosarcomas (CCBC). Aim of this study was to assess the frequency and prognostic impact of *IDH* mutations in high grade CCBC patients.

**Methods:**

64 patients with G2 and G3 CCBC were included. DNA extraction, PCR amplification of *IDH1/2* exon 4s, and sequencing analysis with Sanger were performed.

**Results:**

*IDH* mutations were detected in 24/54 patients (44%): *IDH1* in 18, *IDH2* in 4, and both *IDH1/2* in 2 patients. The frequency of mutations was 37% in G2 vs. 69% in G3 (p = 0.039), and 100% in three Ollier disease associated chondrosarcoma. 5-year overall survival (OS) at 124 months (range 1-166) was 51%, with no significant difference based on the *IDH* mutational status: 61% in *IDHmut* vs. 44% in *IDH* wild type (*IDHwt*). The 5-year relapse free survival (RFS) was 33% (95% CI:10–57) for *IDHmut* vs. 57% (95%CI: 30–77) for *IDHwt*. Progression free survival (PFS) was 25% (95%CI:1–65) *IDHmut* vs. 16% (95%CI: 0.7–52) *IDHwt*. 55% (5/9) of *IDHmut* G2 became higher grade at the recurrence, as compared with 25% (3/12) of G2 *IDHwt.*

**Conclusions:**

This study shows a higher frequency of *IDH* mutations in G3 CCBC as compared with G2. No significant differences in OS, RFS, and PFS by mutational status were detected. After relapse, a higher rate of G3 for *IDH* mutated CCBC was observed.

## Background

Cartilaginous bone sarcomas are the second most frequent bone tumors in adults with roughly 3 new cases per 10^6^ population per year, while in children and young adults they represent only 3% of bone malignancies [1]. They include different histologic subtypes, all characterized by the production of a hyaline cartilaginous matrix, and conventional chondrosarcoma is the most common (85–90%) [2]. Conventional central bone chondrosarcoma (CBCC) arises from the inner bone, while peripheral chondrosarcoma from the surface. Both can rise as primary, de novo, lesions or from the transformation of pre-existing benign lesions, through mutations in different genes: central chondrosarcoma from enchondroma (*IDH mutations*), peripheral from osteochondroma (*EXT1, EXT2 mutations*) [3]. Dedifferentiated chondrosarcoma arises from conventional chondrosarcoma and behaves more aggressively; in the last WHO classification [2] it is considered a separate high-grade chondrosarcoma subtype, defined by a bimorphic histological appearance of a conventional chondrosarcoma component and a high-grade non-cartilaginous sarcoma (more often undifferentiated pleomorphic sarcoma or osteosarcoma, rarely it demonstrates features of angiosarcoma, leiomyosarcoma, rhabdomyosarcoma, or shows epithelial differentiation) [2]. The cartilaginous and sarcomatous components are juxtaposed, generally with an abrupt transition between them, and *IDH* mutations have been described in both components. In the largest series reported in literature, the percentage of dedifferentiation occurring in conventional chondrosarcomas is around 15% [2, 4–6].

Tumor grade is the main prognostic factor. It is defined by cellular atypia, mitoses, and cellularity [7]. Metastasis occurs exceptionally in grade 1 lesions, 10% in grade 2, but up to 70% in grade III lesions [7]. The 5-year overall survival (OS) is 80–90% for grade I but drops to about 50–60% for grade II-III conventional chondrosarcoma [8–10].

Surgery is the cornerstone therapy. Chemotherapy has limited efficacy in CCBC [11–13], comparing with dedifferentiated chondrosarcoma [14], the response rate in CCBC is low. [15, 16]. Novel therapeutic approaches urge the understanding of underlying pathogenetic mechanisms [17–19].

Somatic mutations in the Krebs cycle enzyme isocitrate dehydrogenase (*IDH*) have been described in gliomas, acute myeloid leukemia, cholangiocarcinoma, melanoma, colorectal, prostate cancer, and thyroid carcinoma [20]. In mesenchymal tumors, *IDH* mutations are present in about half of central cartilaginous tumors: in 52% of central low-grade, 58.9% of G2-G3 CCBC, and 56.5% of dedifferentiated chondrosarcoma [5]. Patients with Ollier disease and Maffucci syndrome carry *IDH* mutations with evidence of somatic and intraneoplastic mosaicism (mutation in 81% Ollier e 77% Maffucci) [21, 22].

Biological mechanisms by which cancerogenesis is induced by mutant *IDH* are to be fully elucidated. Mutant isoforms 1 and 2 of *IDH* form with the wild-type protein a heterodimeric enzyme that gains a neomorphic activity, which consists in the NADPH dependent reduction of α-ketoglutarate to D-2-hydroxyglutarate (D-2-HG). D-2-HG behaves as an oncometabolite, competitively inhibiting α-Kg-dependent dioxygenases, inducing DNA methylation rewiring, HIF1α a stabilization independently of oxygen levels, and alteration of the cellular redox balance [23–26].

*IDH* prognostic role is controversial in different reported series of chondrosarcoma patients [27–33] However, a limitation of most of these studies is the inclusion of patients with different histological types and different histological grade [34]. In addition, some of these studies are multicentric [34]. The aim of our study is to assess the frequency of *IDH* mutations, their relationship with clinical characteristics, and their prognostic role in patients with high grade CBCC treated in the same institution.

## Methods

### Study design

The aim of the study was to describe the frequency and type of *IDH* mutations in G2 and G3 CBCC and identify correlations with clinical characteristics and outcome. Inclusion criteria: surgery of primary tumor at our institution from 2002 to 2012, confirmed diagnosis of G2 or G3 conventional chondrosarcoma, availability of both paraffin-embedded and fresh frozen tissue samples. A comprehensive written informed consent was signed for the surgical procedure and related diagnostic procedures in accordance with the standard institutional procedure. After Ethical Institutional Committee approval, clinical data on the diagnosis, treatments, and follow-up (according to institutional guidelines) were collected retrospectively from the patient charts. Surgical margins were defined according to the Enneking score system [33].

Molecular analyses were performed on formalin-fixed and paraffin-embedded tissues (FFPET) and/or frozen tumor samples of patients included in the study. All information regarding the human material was managed using anonymous numerical codes, clinical data were not used, and samples were handled in compliance with the Helsinki declaration.

### DNA extraction

Total DNA extraction from FFPET samples was performed as previously described [33]. A representative tumor area selected on hematoxylin-eosin by the pathologist, which has more than 80% tumor cells, was selected on paraffin block using Multi-Purpose Sampling Tool (Harris UNI-CORE, TedPella Inc, USA) which allows to take tissue cores from the sample, purifying DNA from two cores for each sample. With some modifications, the same QIAamp FFPE Tissue kit (Qiagen) was used for DNA purification of frozen samples. In brief, a fragment of less than 10 mg of tumor tissue was selected using scalpel blades. For each sample 180 µl of lysis buffer and 20 µl of Proteinase K Solution were added. After mechanical stirring, samples were incubated in a water bath overnight at 56 °C. Hematoxylin-eosin cryostat sections were performed to evaluate if the area of tissue selected was representative of the tumor. Quantitative and qualitative analysis was carried out on DNA obtained from both FFPET and frozen tissue by measuring the absorbance (A) in a spectrophotometer to determine the concentration and purity. DNA was considered suitable for molecular analysis only if the A260/A280 ratio was greater than 1.8.

### Sequencing of polymerase chain reaction products

The PCR conditions have been previously described [33]. The Sanger sequencing was performed by Bio-Research Fab (www.biofabresearch.it, Rome, Italy). Mutation analysis was conducted with Basic Local Alignment Search Tool (BLAST), in the NCBI database “National Center of Biotechnology Information Database” (http://www.ncbi.nlm.nih.gov/BLAST). Electropherograms were exported to fasta format and were aligned to the NCBI BLAST sequences.

### Statistical analysis

Continuous variables were summarized as mean and range; discrete and categorical variables were summarized using frequencies and percentages. Patient characteristics at diagnosis (age, site, grade, margins according to Enneking [35], and stage), were compared between *IDH* mutation using Chi-Square test. OS was defined as the time from the date of surgery to the date of death. Patients who did not experience the outcome of interest were censored at the time of last follow-up. RFS was calculated in localized patients and was defined as the time from date of surgery to the date of first relapse. PFS was calculated in metastatic patients and was defined as the time from date of surgery to the date of progression. Patients who died for surgery complications were excluded from survival analysis. Kaplan-Meier methods were used to estimate OS, RFS and PFS and the curves were compared using log-rank test. A value of p < 0.05 was considered statistically significant. All p values were 2-sided. Data were analyzed using the SAS 9.4 software (SAS Institute).

## Results

### Frequency and type of mutations

DNA extraction was performed on tumor samples derived from the first surgical procedure in 64 patients included in the protocol.

Molecular analysis was not evaluable in 10 patients, for scarcity of extracted DNA, due to aged slides and decalcification procedures. In 54/64 (84%) cases the quality of tissue was adequate: *IDH* mutations were detected in 24 patients (44%): in 18 *IDH1mut* only (34%), in 4 *IDH2mut* only (7%), and in 2 patients both *IDH1 and 2mut* (3%). *IDH1* was mutated on residue 132 of arginine while *IDH2* on residue 172, in the substrate-binding site. Details on mutation types are described in Table 1 and Fig. 1.

**Table 1:** Distribution of gene mutation types in positive cases (the sum is > 100% since 2 patients had both *IDH1* and *IDH*2 mut)

Gene	Mutation	Prevalence
*IDH1*	R132C (tgt)	63% (15/24)
*IDH1*	R132G (ggt)	17% (4/24)
*IDH1*	R132S (agt)	4% (1/24)
*IDH2*	R172S (agt)	17% (4/24)
*IDH2*	R172S (agc)	4% (1/24)
*IDH2*	R172G (ggg)	4% (1/24)

**Fig. 1a:**
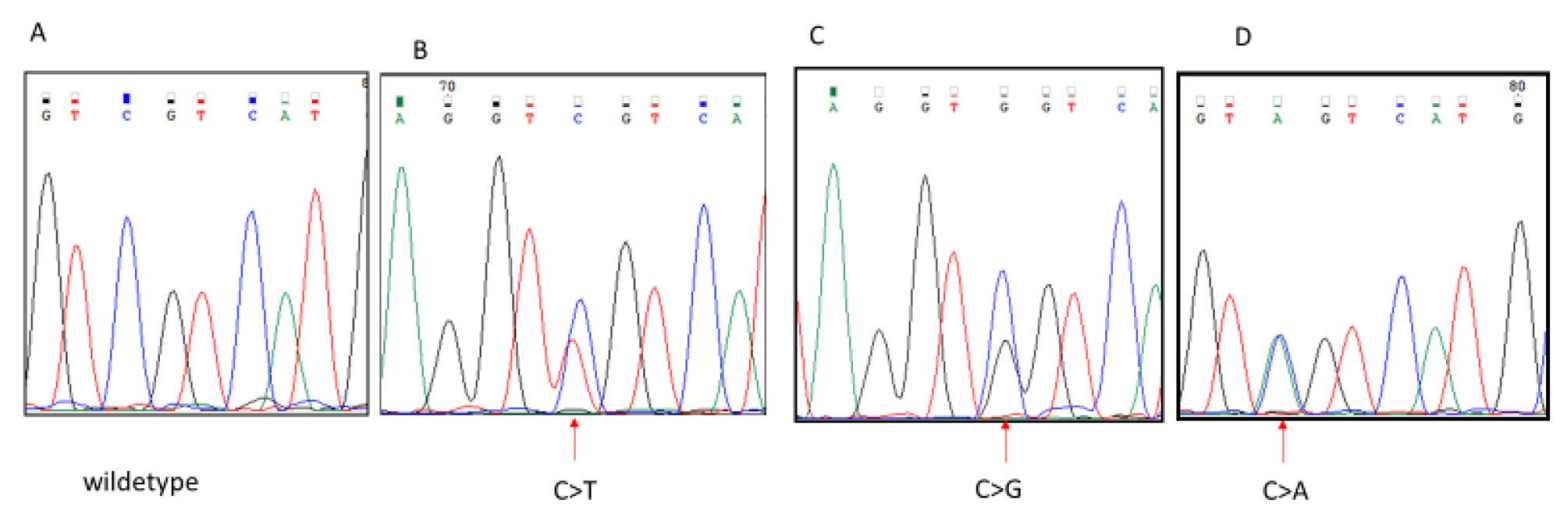
Sanger sequencing performed on IDH1 gene, exon 4. **(A)** Electropherogram data from a wild-type sequence in one of the tested samples; **(B)** chondrosarcoma sample harboring a R132C IDH1 (arrow) mutation (c.394 C>T); **(C)** chondrosarcoma sample harboring R132G (arrow) mutation (c.394 C>G); **(D)** sample harboring R132S (arrow) mutation (c.394 C>A)

**Fig. 1b:**
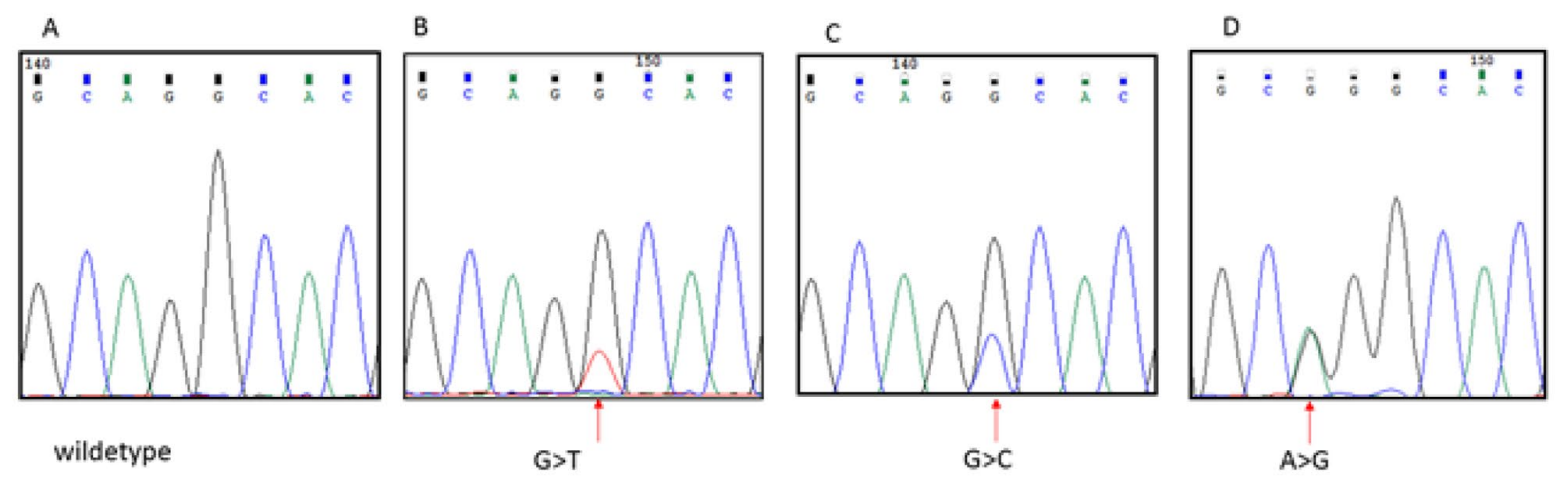
Sanger sequencing performed on IDH2 gene, exon 4. **(A)** Electropherogram data from a wild-type sequence in one of the tested samples; **(B)** chondrosarcoma sample harboring a R172S (arrow) mutation (c.516 G>T); **(C)** chondrosarcoma sample harboring R172S (arrow) mutation (c.516 G>C); **(D)** sample harboring R172G (arrow) mutation (c.514 A>G)

**Fig. 2:**
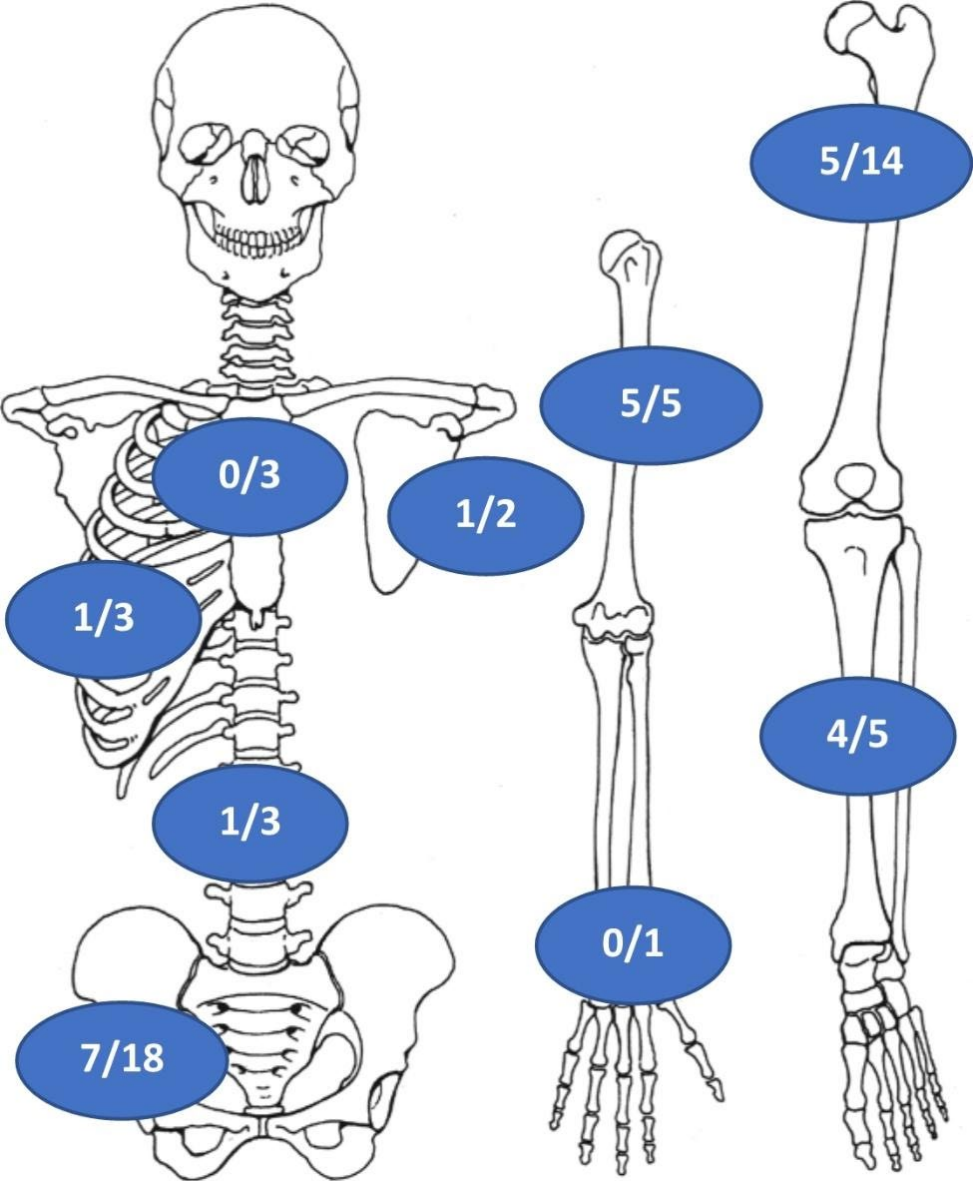
Frequency of mutations by site. Humerus and tibia were the sites with higher mutation rate, followed by femur and pelvi. One *IDHmut* vertebral CCBC has been found

### Patients’ clinical characteristics by mutational status

Of the 54 analyzed patients 18 (33%) were males and 36 (67%) females; the median age was 63 (range 17–85 years). Distribution by site is illustrated in Fig. 2.

**Table 2:** Patient characteristics according to *IDH* mutation. Median age was comparable in mutated and wild-type groups. The IDH mutation rate was not different in localized (43%) vs. metastatic (50%) patients; while it was significantly higher in G3 (69%) than in G2 (37%) tumors (p=0.0390)

	all sample = 54	*IDH*mut N = 24	*IDH*wt N = 30	P value
**Age**, median(range)	63 (17–85)	60 (33–85)	64 (17–79)	0.2889
**Site, n (%)**				
Extremity	25 (46%)	14 (56%)	11 (44%)	0.2357
Pelvis	18 (33%)	7 (39%)	11 (61%)	
Other	11 (21%)	3 (27%)	8 (73%)	
**Grade, n (%)**				
G2	41 (76%)	15 (37%)	26 (63%)	0.0390
G3	13 (24%)	9 (69%)	4 (31%)	
**Stage, n (%)**				
Localized	40 (74%)	17 (43%)	23 (57%)	0.6269
Metastatic	14 (26%)	7 (50%)	7 (50%)	
**Margins, n (%)**				
Wide	39 (75%)	17 (44%)	22 (56%)	0.5206
Not-wide	13 (25%)	7 (54%)	6 (46%)	

**Table 3:** Patient characteristics according to *IDH* mutation split by grade. No differences were found between patients with *IDHmut* and *IDHwt* tumors in terms of localization, stage at diagnosis, morphology, and Syndrome coexistence

	G2 (N = 41)				G3 (N = 13)			
	*IDH*mut N = 15 (37%)	*IDH*wt N = 26 (63%)	All G2	P value	*IDH*mut N = 9 (69%)	*IDH*wt N = 4 (31%)	All G3	P value
**Site**, n (%)								
Extremity	10 (67)	10 (38)	20	0.23	4 (44)	1 (25)	5	0.44
Pelvis	3 (20)	10 (38)	13		4 (44)	1 (25)	5	
Other	2 (13)	6 (24)	8		1 (12)	2 (50)	3	
**Stage**, n (%)								
Localized	12 (80)	21 (81)	33	1.0	5 (56)	2 (50)	7	1.0
Metastatic	3 (20)	5 (19)	8		4 (44)	2 (50)	6	
**Margins**, n (%)								
Wide	10 (67)	20 (83)	30	0.27	7 (78)	2 (50)	9	0.53
Not-wide	5 (33)	4 (17)	9		2 (22)	2 (50)	4	
Uk		2	2					

At the time of surgery 40 patients (74%) presented with localized disease, and 14 (26%) with lung metastases. 41 patients had G2 chondrosarcoma at pathological examination, while 13 had G3 tumors. 8 (19%) G2 patients, and 6 (42%) G3 patients presented with metastases.

A comparison of clinical characteristics between *IDHmut* and *IDHwt* cohorts is illustrated in Table 2. On histological examination, no different morphological features have been reported for mutant versus wild-type tumors.

As expected, syndromic conditions were confirmed to be a substrate for *IDHmut* conventional chondrosarcoma. The three chondrosarcomas of patients with Ollier syndrome (100%) harbored a mutation of *IDH1*, and all 3 cases shared the same mutation: R132C(TGT); none of the patients presented *IDH2* mutation. One patient with Maffucci’s syndrome had a metacarpal chondrosarcoma *IDH1wt* and *IDH2* not evaluable.

In Table 3 the comparison between G2 and G3 groups is reported.

### Evolution/dedifferentiation

We observed a different grade progression over time (at relapse) in the 2 groups (*IDH1/2mut* or *wt*): *IDHmut* chondrosarcoma had a higher rate of grade progression at relapse, as compared to *IDHwt*. In particular 25 patients (21 cases presenting with G2 at diagnosis and 4 presenting with G3) had a new histologic evaluation at recurrence/progression: 5/9 (55%) of G2 *IDH1/2mut* tumors became higher grade at recurrence (1 became G3 and 4 dedifferentiated); while only 3/12 (25%) of G2 *IDH1/2wt* acquired a higher grade in the recurrence (3 became dedifferentiated).

Two *IDH1wt* patients developed second malignancies: 1 desmoid fibromatosis (and had had a previous prostatic adenocarcinoma) and 1 non-Hodgkin lymphoma.

Two patients with *IDHmut* developed a second malignancy: 1 prostatic adenocarcinoma and 1 patient with Maffucci Syndrome developed second chondrosarcoma at a different site. Three *IDHmut* patients with Ollier disease, had evidence of pre-existing enchondroma. Moreover, one *IDH1/2* patient had evidence of enchondroma, G2 chondrosarcoma, and dedifferentiated chondrosarcoma recapitulating in his history all the phases of cancerogenesis.

### Treatment

Surgery consisted o: 42 resections, 11 amputations, and 1 curettage. Wide margins were obtained in 39 pts. None of the 40 cases with localized disease received adjuvant chemotherapy. 6/14 patients metastatic at diagnosis (4 *IDHmut*, 2 *IDHwt*) underwent first line chemotherapy.

Treatment at recurrence/progression consisted of surgery (19 patients) chemotherapy (9 patients), and radiotherapy (8 patients).

**Fig. 3:**
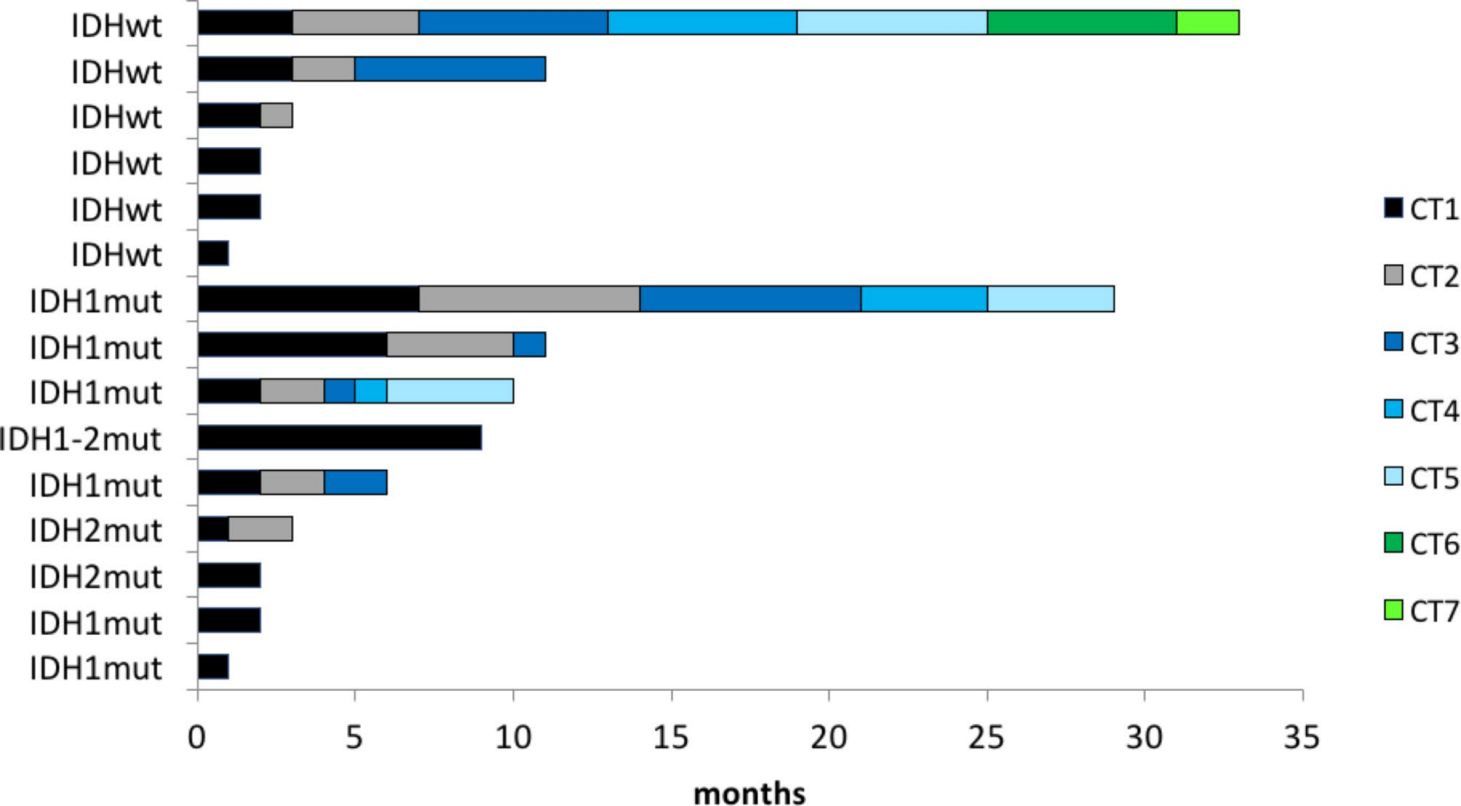
Chemotherapy lines and their duration. Number of chemotherapy (CT) lines in 15 patients treated with chemotherapy at recurrence/progression of disease, and duration in month for each CT line, by mutational status. Only the patient with IDH1/2 mut who received chemotherapy according to Euro-B.O.S.S. protocol [11] for a dedifferentiated recurrence, had tumor shrinkage. One patient received adjuvant chemotherapy after dedifferentiated recurrence. The other 13 patients had disease progression after 2-6 months in all chemotherapy lines: 5 of them received rapamycin and cyclophosphamide (but only one achieved a disease stabilization as best response) and 2 with hedgehog inhibitors

Number and duration of chemotherapy lines are summarized in Fig. 3.

### Outcome

Survival analysis was performed on 50 patients: 4 patients died of surgical complications and were excluded from the outcome analysis. After a median follow-up of 124 months (range 1-166) relapse occurred in 21/40 localized patients (10 *IDHmut*): 11 local recurrences, 5 lung metastases, 1 lymph node metastases, and 4 both local relapse and metastasis. Disease progression was reported for all metastatic patients.

The 5-years OS was 51% (95% CI:36–64), and was significantly higher in patients with localized vs. metastatic disease at diagnosis (69% vs. 7%, p < 0.001), and in patients with G2 vs. G3 (59% vs. 29%, p = 0.0078) chondrosarcoma.

The 5-years OS in *IDHmut* vs. *IDHwt* was not significantly different in the general population, nor in the split group of G2 and G3, as reported in Fig. 4a, 4b, 4c. No differences between *IDHmut* and *IDHwt* in the 5-years RFS and PFS for localized and metastatic patients respectively has been detected, as reported in Fig. 5a and 5b).

**Fig. 4a:**
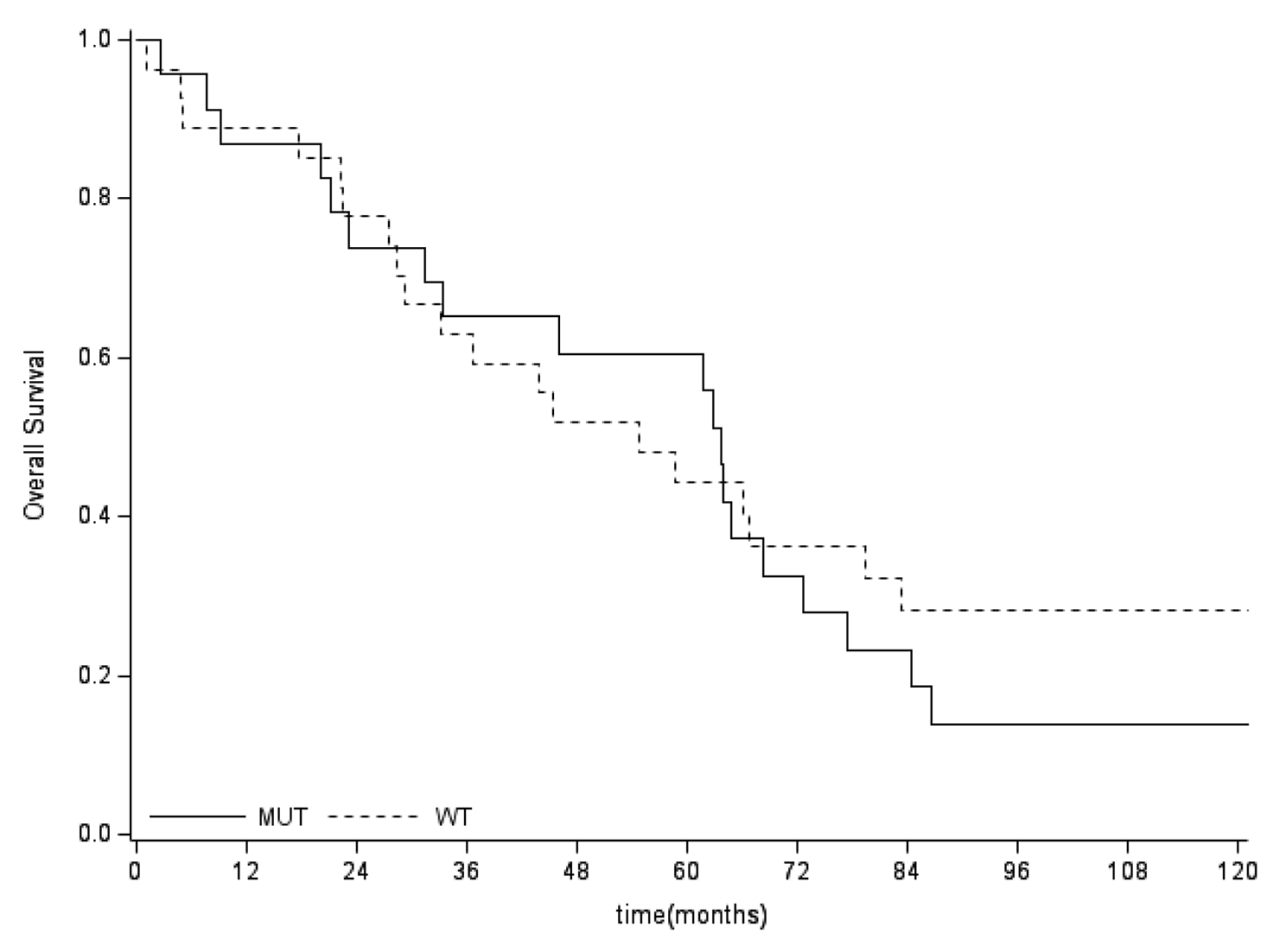
Overall survival according to mutational status in the general population. No significant difference in survival by mutational status (5-years OS *IDHmut* 61% vs *IDHwt* 44%, p=0.6854) was detected

**Fig. 4b:**
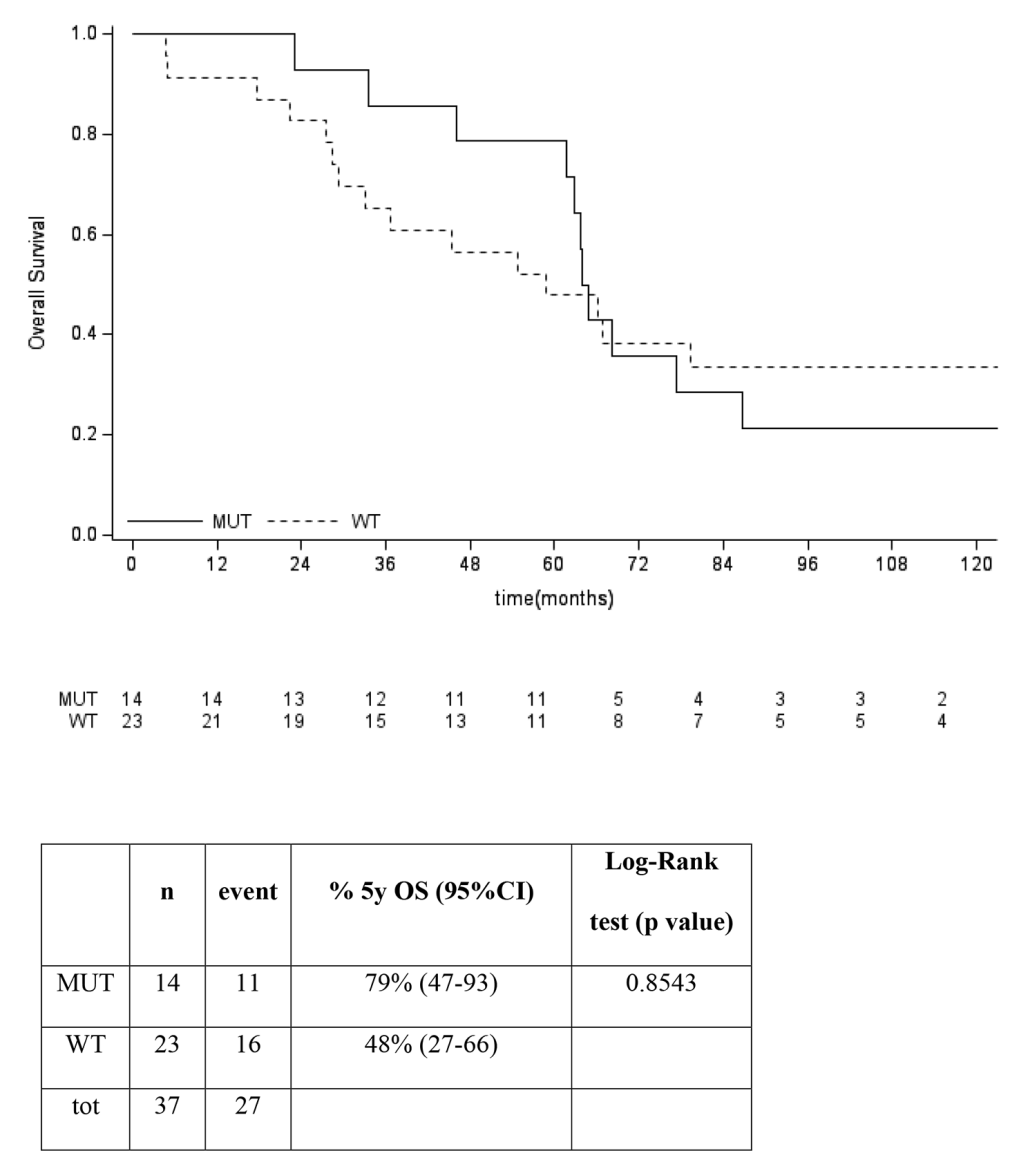
Overall survival according to mutational status in G2 patients. 5-years OS in G2 was 79% (95% CI: 47-93) for *IDHmut* vs 48% (95% CI: 27-66) for *IDHwt* (p 0.8543)

**Fig. 4c:**
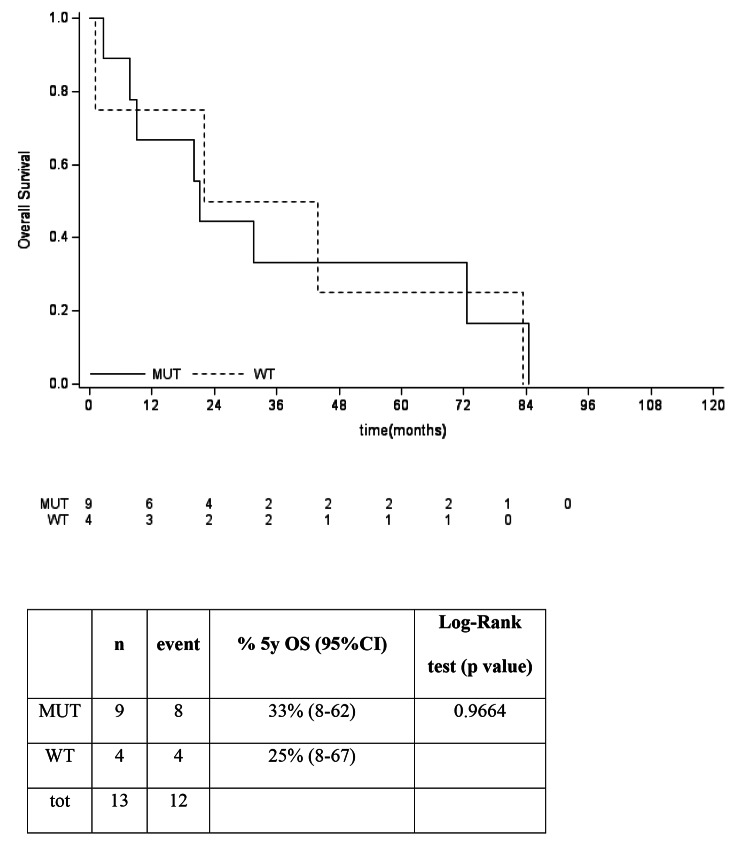
Overall survival according to mutational status in G3 patients. For patients with G3 CCBC 5y OS was 33% (95% CI 8-62) in *IDHmut* vs 25% (95% CI: 8-67) in *IDHwt* (p 0.9664)

**Fig. 5a:**
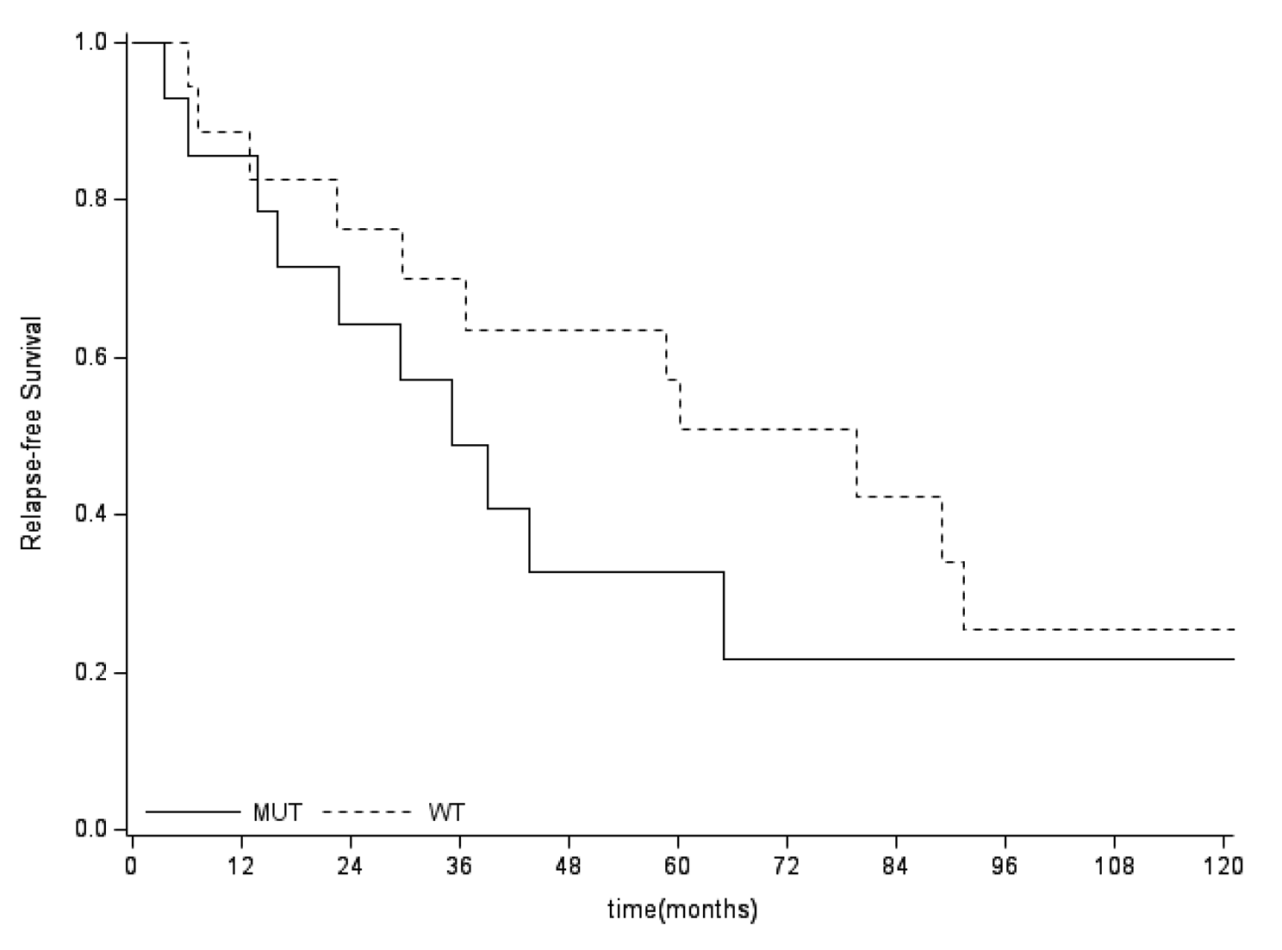
Relapse-free survival according to mutational status (localized patients). The 5-years RFS in localized patients was 33% (95% CI:10-57) in *IDHmut* and 57% (95%CI: 30-77) in *IDHwt* (p=0.3596)

**Fig. 5b:**
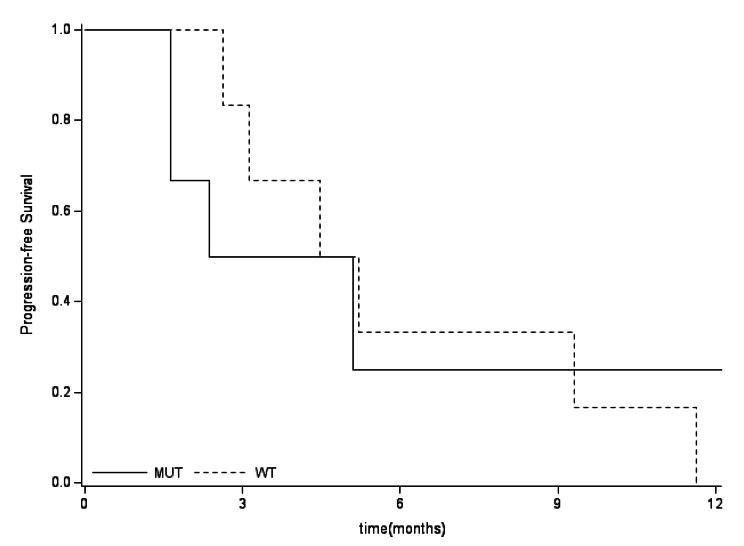
Progression-free survival by mutational status (metastatic patients). In patients presenting with metastases, the PFS was 25% (95%CI: 1-65) in *IDHmut* and 16% (95%CI: 0.7-52) in *IDHwt*, (p=0.9126)

## Discussion

*IDH* mutations were first identified in 2006 in colon cancer [36], and next in many other tumor types, but only in glioma a clear correlation between genetic alteration and disease phenotype and prognosis has been established [37].

Amary and Pansuriya pioneering works show that *IDH* mutations in mesenchymal tumors: 1- are prevalent in cartilaginous tumors rather than in other connective tissue sarcomas; 2- are present in 80% of benign enchondromas and in almost half of conventional chondrosarcomas; 3- occur early in the tumorigenesis; 4- both local and distant recurrences of central conventional chondrosarcoma maintain the same IDH mutational status of the primary lesion; 5- are almost always present in enchondromas of Ollier and Maffuci syndromes [21, 22, 38].

Other authors investigated on the presence and role of *IDH* mutations in chondrosarcomas, but most of the reported series include benign with high grade, tumors from different sites, and also different chondrosarcoma histotypes. In these series the number of G2 and G3 chondrosarcoma account from 24 to 51. [27–34]. In the current study, we decided to focus on G2-3 CCBC, in order to have a homogeneous population, reporting data on 54 patients all treated in one institution.

Although the use of sole Sanger sequencing detects somatic variants at frequency levels above 10% [39], and might lead to false-negative *IDHwt* cases, the results obtained were consistent with literature data: the type of mutations (R132 in *IDH1* and R172 in *IDH2*, but not R140 in *IDH2*), the frequency (44%) and the *IDH1* to *IDH2* mutation ratio 20:6. In a future work it would be possible to confirm the wt cases with a more sensitive technique such as NGS.

The *IDH* mutation rate was significantly higher in G3 (69%) than in G2 (37%) tumors (p = 0.0390). At recurrence, CBCC is estimated to exhibit a higher grade of malignancy in more than 10% of cases, but the biological mechanisms involved in the progression are still unknown [9]. We observed a higher rate of grade progression at recurrence in *IDHmut* CBCC: 55% *IDHmut* vs. 25% *IDH wt* G2 became higher grade at relapse. One patient with *IDH1/2mut* G2 had evidence of pre-existing enchondroma, and developed a dedifferentiated chondrosarcoma. Taken together, our data suggest that *IDH* mutation is one of the events responsible for low to high grade progression, and that *IDH* mutant cells are more susceptible to changes toward dedifferentiation. It’s reported that p16/CDKN2A copy number variation, in *IDHmut* CBCC, occurs only after the onset of *IDH* mutation [38], and that the methylation status changes from low grade and high grade *IDHmut* chondrosarcoma. [40, 41].

The impact of *IDH* mutation on chondrosarcoma prognosis is still unclear. Stage, grade, size, and surgical margins are the main factors to consider for survival prediction in CCBC. In our series grade and stage were confirmed to be prognostic, but not the mutational status. The 5-year OS was 51%, but dropped to 7% in metastatic patients, and to 29% in G3 patients, but did not differ significantly by mutational status. The 5-year RFS was worse in *IDHmut* localized patients, but not statistically significant. Similarly, Lugowska et al. [27] found a shorter survival in *IDH1/2mut* patients, but in that series different histotypes other than CBCC have been included, while Zhou et al. [28] reported longer RFS and metastasis-free survival (MFS) in *IDHmut* CCBC (51 G2-3), but did not detect a significant impact of mutational status on OS. Cleven et al. [29], and Amary et al. [5] could not find differences in DFS and MFS, and survival period respectively. A meta-analysis using individual patient data from 14 studies found a significant negative impact of *IDH1/2* mutations on patient survival, but not on RFS and MFS [34]. Possible explanations for the discrepancies in defining *IDH* mutations prognostic role between available studies could in part rely on the rarity of the disease (small sample size), heterogeneity of included population (patients with different chondrosarcoma subtypes and different grade), the employment of different sequencing tools. Moreover, even if *IDH* mutation is considered one of the most important drivers of chondrosarcoma, other genes seem to be involved in tumor progression (p53, Rb, histone deacetylase, and others) and may impact on patient prognosis.

Target therapies with potential activity in chondrosarcoma include antiangiogenic agents, mTOR inhibitors, hedgehog inhibitors, histone deacetylase inhibitors, immunotherapy, and, more recently, IDH inhibitors. [18, 19]. IDH inhibitors are approved for the treatment of *IDHmut* acute myeloid leukemia. Ivosidenib, a first-in-class selective oral IDH1 inhibitor, has shown clinical activity in preclinical studies [42, 43] and in a phase 1, open-label, multicenter study with 168 patients with advanced solid tumors. Chondrosarcoma dose-escalation and expansion cohorts included 21 patients (14 with G2-G3 CCBC) with recurrent/progressing disease: 11 (52%) achieved stable disease, and 4 of them for more than 2.5 years [43]. Ongoing trials are still recruiting chondrosarcoma patients [19] to understand the more appropriate setting for IDH1/2 inhibitors.

## Conclusion

No different clinical characteristics were found between *IDHwt* and *IDHmut* high grade CCBC, except a higher frequency of mutations in syndromic patients and in G3 lesions. No statistically significant difference in prognosis was detected between *IDHwt* and *IDHmut* A different trend in histologic evolution was detected, with *IDHmut* G2 tumors having a higher rate of grade progression at relapse, as compared to *IDHwt*.

## Data Availability

The Sanger sequencing data analyzed during the current study have been deposited in Genome Sequence Archive for Human and made available publicly with the accession number: HRA003467. at https://ngdc.cncb.ac.cn/gsa-human.

## Declarations

Comprehensive written informed consent was signed for the surgical procedure and related diagnostic procedures in accordance with the standard institutional procedure.

All information regarding the human material was managed using anonymous numerical codes, clinical data were not used, and samples were handled in compliance with the Helsinki declaration.
